# Supplementary material for: Synchronous Ovarian Sertoli–Leydig Cell and Clear Cell Papillary Renal Cell Tumors: A Rare Case Without Mutations in Cancer-Associated Genes
Source: Curr Oncol. 2025 Jul 30;32(8):429. doi: 10.3390/curroncol32080429 (PMC12384082; doi:10.3390/curroncol32080429)
Supplement: Supplementary file 1 [file curroncol-32-00429-s001.zip › curroncol-3682794-supplementary.pdf]

Supplementary details on Molecular diagnosis

a) *Whole exome sequencing and Bioinformatics Analysis*

DNA quantification was performed using a Qubit 3.0 Fluorometer with the Qubit dsDNA HS (High Sensitivity) Assay Kit fluorescent dye method. According to the manufacturer's instructions, we used 100 ng of DNA for Whole Exome Sequencing (WES). Libraries were prepared using the Kapa HyperPlus kits (Roche Molecular Systems Inc., Santa Clara, CA, USA). Sequencing was performed using NovaSeq 6000 (Illumina Inc., San Diego, CA, USA) with an average sequencing depth of at least 100x. To process the WES data and to perform variant calling and variant annotation, the sequences were aligned to the human reference genome GRCh37 (http://www.ncbi.nlm.nih.gov/projects/genome/assembly/grc/human/index.shtml, accessed October 11, 2018) using the Burrows-Wheeler Aligner with default parameters. Trimming, base calling, coverage analysis and variant calling were performed using an in-house bioinformatics pipeline (bcl to fastq version 2.20, Isaac Aligner version 4, GATK "Genome Analysis Toolkit" version 4, Sam tools version 1.9 and Bed tools version 2). Vcf analysis was performed using the Illumina Variant Interpreter interpretation and reporting platform, filtered by DICER1 gene, by quality > 15, by small variant consequences such as stop gains, splice donors, splice acceptors, splice region, frameshift indels, in-frame deletions, in-frame insertions, initiator codon (ATG) losses, missense protein changes, incomplete terminal codon. It was additionally filtered for frequencies < 0.05 in European populations using tools such as the 1000 Genomes Project (https://www.internationalgenome.org/, accessed October 11, 2018), gnomAD (https://gnomad.broadinstitute.org/), Exome Aggregation Consortium (http://exac.broadinstitute.org/, accessed October 11, 2018) and Human Gene Mutation Database (HGMD, http://www.hgmd.cf.ac.uk/ac/, (accessed October 11, 2018). We only selected variants affecting coding exons or canonical splice sites. Variants were classified according to the guidelines of the American College of Medical Genetics and Genomics (ACMG). Finally, synonymous variants were filtered out to detect only rare variants (frequency of <0.1%) in both dbSNP and our in-house database containing >1000 exomes) with high quality.

The analysis of patients' genomic DNA was performed using high depth NGS, expanding the study to a panel of approximately 280 genes that, when mutated, are associated with predisposition to various hereditary and sporadic cancers (Table S1). This approach allows the identification of pathogenic or likely pathogenic mutations in 8-15% of cases, thus increasing the diagnostic, prognostic and therapeutic value of next-generation sequencing in oncology.

The variants in each of the listed genes were interpreted by importing the ".vcf" files obtained with two different variant callers (Isaac and GATK) into the Illumina Variant Interpreter analysis and

prioritization tool. This tool was set to filter by functional consequences (including stop gain, stop loss, splice donor, splice acceptor, splice region, frameshift indels, inframe deletions, inframe insertions, ATG loss in initiator codon, missense, protein modification, incomplete terminal codon) and frequency (< 0.5 in population frequency databases such as GnomAD, GnomADExome, 1000 Genome Project, NHLBI Exome Sequencing Project), and specific metrics (e.g., quality > 15).

Based on these criteria, no changes were detected in any of the genes examined, with the exception of the variant c.1814C>G p.(Thr605Ser, rs587781616, exon 4/10) in the MSH6 gene (NM_000179.2). This variant is listed in the dbSNP database and annotated in ClinVar as a variant with conflicting interpretation of pathogenicity, which has been described 9/10 times as a variant of unknown significance (VUS) for Lynch syndrome and hereditary cancer predisposition.

b) MLPA (Multiple Ligation-dependent Probe Amplification reaction)

As indicate in the text, most of the mutations described in DICER1 are point or frameshift mutations with loss of function. However, deletions of the entire DICER1 locus and in- or out-of-frame intragenic DICER1 deletions have also been reported (20).

For this reason, DICER1 dosage analysis was performed in the proband using the Multiple Ligation-dependent Probe Amplification reaction (MLPA), a test to detect deletions or duplications in the target genes. The assay was performed using SALSA P482-A1 (MRC-Holland, Amsterdam, The Netherlands), which contains 50 MLPA probes with amplification products ranging from 124 to 500 nucleotides (nt), including 31 probes for the DICER1 gene, 4 probes centromeric and 3 probes telomeric to the DICER1 gene, and an additional 12 reference probes targeting relatively copy number stable regions in various cancer types. This probe mix also contains nine quality control fragments that generate amplification products between 64 and 105 nt: four DNA Quantity fragments (Q fragments), two DNA Denaturation fragments (D fragments), one Benchmark fragment, and one chromosome X-specific and one chromosome Y-specific fragment (https://www.mrcholland.com/product/P482). The reactions were performed according to the manufacturer's recommendations (100 ng DNA/reaction, hybridization time of 16 hours and 35 PCR cycles). Reaction products were separated using the ABI Prism 3500 Genetic Analyzer, (Applied Biosystems, Thermos Fisher Scientific, Waltham, MA, USA). Analysis was performed using Coffalyser.Net software (from MRC-Holland, downloadable at www.mrcholland.com) with default parameters and dose ratio limits < 0.80 and > 1.20 for deletion and duplication, respectively.

Supplementary Table
List of the genes potentially associated to tumors and screened for mutation.

Type of tumors	Gene name	Protein
Not specified	AIP	Aryl hydrocarbon receptor interacting protein
	ALK	Anaplastic lymphoma kinase
	APC	Adenomatous polyposis coli
	APEX1	APEX nuclease (multifunctional DNA repair enzyme) 1
	ATM	Ataxia telangiectasia mutated
	ATMIN	ATM interactor
	ATR	Ataxia telangiectasia and Rad3 related
	ATRIP	ATR interacting protein
	AURKA	Aurora kinase A
	AXIN1	Axin 1
	BABAM1	BRISC and BRCA1 A complex member 1
	BAP1	BRCA1 associated protein-1 (ubiquitin carboxy-terminal hydrolase)
	BARD1	BRCA1 associated RING domain 1
	BLM	Bloom syndrome, RecQ helicase-like
	BMPR1A	Bone morphogenetic protein receptor, type IA
	BRAP	BRCA1 associated protein
	BRCA1	Breast cancer 1, early onset
	BRCA2	Breast cancer 2, early onset
	BRCC3	BRCA1/BRCA2-containing complex, subunit 3
	BRE	Brain and reproductive organ-expressed (TNFRSF1A modulator)
	BRIP1	BRCA1 interacting protein C-terminal helicase 1
	BUB1B	Budding uninhibited by benzimidazoles 1 homolog beta (yeast)
	C11orf30	Chromosome 11 open reading frame 30 (EMSY)
	C19orf40	Chromosome 19 open reading frame 40 (FAAP24)
	CASP8	Caspase 8, apoptosis-related cysteine peptidase
	CCND1	Cyclin D1
	CDC73	Cell division cycle 73, Paf1/RNA polymerase II complex component, homolog (S. cerevisiae)
	CDH1	Cadherin 1, type 1, E-cadherin (epithelial)
	CDK4	Cyclin-dependent kinase 4
	CDKN1B	Cyclin-dependent kinase inhibitor 1B (p27, Kip1)
	CDKN1C	Cyclin-dependent kinase inhibitor 1C (p57, Kip2)
	CDKN2A	Cyclin-dependent kinase inhibitor 2A
	CEBPA	CCAAT/enhancer binding protein (C/EBP), alpha

	CEP57	Centrosomal protein 57kDa
	CLSPN	Claspin
	CSNK1D	Casein kinase 1, delta
	CSNK1E	Casein kinase 1, epsilon
	CWF19L2	CWF19-like 2, cell cycle control (S. pombe)
	CYLD	Cylindromatosis (turban tumor syndrome)
	DCLRE1C	DNA cross-link repair 1C
	DDB2	Damage-specific DNA binding protein 2, 48kDa
	DHFR	Dihydrofolate reductase
	DICER1	Dicer 1, ribonuclease type III
	DMC1	DMC1 dosage suppressor of mck1 homolog, meiosis-specific homologous recombination (yeast)
	DNAJC21	DnaJ (Hsp40) homolog, subfamily C, member 21
	DPYD	Dihydropyrimidine dehydrogenase
	EGFR	Epidermal growth factor receptor
	EPCAM	Epithelial cell adhesion molecule
	EPHX1	Epoxide hydrolase 1, microsomal (xenobiotic)
	ERCC1	Excision repair cross-complementing rodent repair deficiency, complementation group 1
	ERCC2	Excision repair cross-complementing rodent repair deficiency, complementation group 2
	ERCC3	Excision repair cross-complementing rodent repair deficiency, complementation group 3
	ERCC4	Excision repair cross-complementing rodent repair deficiency, complementation group 4
	ERCC5	Excision repair cross-complementing rodent repair deficiency, complementation group 5
	ERCC6	Excision repair cross-complementing rodent repair deficiency, complementation group 6
	ESR1	Estrogen receptor 1
	ESR2	Estrogen receptor 2 (ER beta)
	EXO1	Exonuclease 1
	EXT1	Exostosin 1
	EXT2	Exostosin 2
	EYA2	Eyes absent homolog 2 (Drosophila)
	EZH2	Enhancer of zeste homolog 2 (Drosophila)
	FAM175A	Family with sequence similarity 175, member A
	FAM175B	Family with sequence similarity 175, member B
	FAN1	FANCD2/FANCI-associated nuclease 1
	FANCA	Fanconi anemia, complementation group A
	FANCB	Fanconi anemia, complementation group B

	FANCC	Fanconi anemia, complementation group C
	FANCD2	Fanconi anemia, complementation group D2
	FANCE	Fanconi anemia, complementation group E
	FANCF	Fanconi anemia, complementation group F
	FANCG	Fanconi anemia, complementation group G
	FANCI	Fanconi anemia, complementation group I
	FANCL	Fanconi anemia, complementation group L
	FANCM	Fanconi anemia, complementation group M
	FBXW7	F-box and WD repeat domain containing 7, E3 ubiquitin protein ligase
	FH	Fumarate hydratase
	FLCN	Folliculin
	GADD45A	Growth arrest and DNA-damage-inducible, alpha
	GATA2	GATA binding protein 2
	GPC3	Glypican 3
	GRB7	Growth factor receptor-bound protein 7
	HELQ	Helicase, POLQ-like
	HNF1A	HNF1 homeobox A
	HOXB13	Homeobox B13
	HRAS	v-Ha-ras Harvey rat sarcoma viral oncogene homolog
	HUS1	HUS1 checkpoint homolog (S. pombe)
	CHEK1	Checkpoint kinase 1
	CHEK2	Checkpoint kinase 2
	KAT5	K(lysine) acetyltransferase 5
	KCNJ5	Potassium inwardly-rectifying channel, subfamily J, member 5
	KIT	Potassium inwardly-rectifying channel, subfamily J, member 5
	LIG1	Ligase I, DNA, ATP-dependent
	LIG3	Ligase III, DNA, ATP-dependent
	LIG4	Ligase IV, DNA, ATP-dependent
	LMO1	LIM domain only 1 (rhombotin 1)
	LRIG1	Leucine-rich repeats and immunoglobulin-like domains 1
	MAX	MYC associated factor X
	MCPH1	Microcephalin 1
	MDC1	Mediator of DNA-damage checkpoint 1
	MDM2	Mdm2, p53 E3 ubiquitin protein ligase homolog (mouse)
	MDM4	Mdm4 p53 binding protein homolog (mouse)

	MEN1	Multiple endocrine neoplasia I
	MET	Met proto-oncogene (hepatocyte growth factor receptor)
	MGMT	O-6-methylguanine-DNA methyltransferase
	MLH1	mutL homolog 1, colon cancer, nonpolyposis type 2 (E. coli)
	MLH3	mutL homolog 3 (E. coli)
	MMP8	Matrix metallopeptidase 8 (neutrophil collagenase)
	MPL	Myeloproliferative leukemia virus oncogene
	MRE11A	MRE11 meiotic recombination 11 homolog A (S. cerevisiae)
	MSH2	mutS homolog 2, colon cancer, nonpolyposis type 1 (E. coli)
	MSH3	mutS homolog 3 (E. coli)
	MSH5	mutS homolog 5 (E. coli)
	**MSH6**	**mutS homolog 6 (E. coli)**
	MSR1	Macrophage scavenger receptor 1
	MUS81	MUS81 endonuclease homolog (S. cerevisiae)
	MUTYH	mutY homolog (E. coli)
	NAT1	N-acetyltransferase 1 (arylamine N-acetyltransferase)
	NBN	Nibrin
	NCAM1	Neural cell adhesion molecule 1
	NELFB	Cofactor of BRCA1
	NF1	Neurofibromin 1
	NF2	Neurofibromin 2 (merlin)
	NFKBIZ	Nuclear factor of kappa light polypeptide gene enhancer in B-cells inhibitor, zeta
	NHEJ1	Nonhomologous end-joining factor 1
	NSD1	Nuclear receptor binding SET domain protein 1
	OGG1	8-oxoguanine DNA glycosylase
	PALB2	Partner and localizer of BRCA2
	PARP1	Poly (ADP-ribose) polymerase 1
	PCNA	Proliferating cell nuclear antigen
	PHB	Prohibitin
	PHOX2B	Paired-like homeobox 2b
	PIK3CG	Phosphatidylinositol-4,5-bisphosphate 3-kinase, catalytic subunit gamma
	PLA2G2A	Phospholipase A2, group IIA (platelets, synovial fluid)
	PMS1	PMS1 postmeiotic segregation increased 1 (S. cerevisiae)

	POLB	Polymerase (DNA directed), beta
	POLD1	Polymerase (DNA directed), delta 1, catalytic subunit
	POLE	Polymerase (DNA directed), epsilon, catalytic subunit
	PPM1D	Protein phosphatase, Mg2+/Mn2+ dependent, 1D
	PREX2	Phosphatidylinositol-3,4,5-trisphosphate-dependent Rac exchange factor 2
	PRF1	Perforin 1 (pore forming protein)
	PRKAR1A	Protein kinase, cAMP-dependent, regulatory, type I, alpha
	PRKDC	Protein kinase, DNA-activated, catalytic polypeptide
	PTEN	Phosphatase and tensin homolog
	PTCH1	Patched 1
	PTTG2	Pituitary tumor-transforming 2
	RAD1	RAD1 homolog (S. pombe)
	RAD17	RAD17 homolog (S. pombe)
	RAD18	RAD18 homolog (S. cerevisiae)
	RAD23B	RAD23 homolog B (S. cerevisiae)
	RAD50	RAD50 homolog (S. cerevisiae)
	RAD51	RAD51 homolog (S. cerevisiae)
	RAD51AP1	RAD51 associated protein 1
	RAD51B	RAD51 homolog B (S. cerevisiae)
	RAD51C	RAD51 homolog C (S. cerevisiae)
	RAD51D	RAD51 homolog D (S. cerevisiae)
	RAD52	RAD52 homolog (S. cerevisiae)
	RAD54B	RAD54 homolog B (S. cerevisiae)
	RAD54L	RAD54-like (S. cerevisiae)
	RAD9A	RAD9 homolog A (S. pombe)
	RB1	Retinoblastoma 1
	RBBP8	Retinoblastoma binding protein 8
	RECQL	RecQ protein-like (DNA helicase Q1-like)
	RECQL4	RecQ protein-like 4
	RECQL5	RecQ protein-like 5
	RET	Ret proto-oncogene
	RFC1	Replication factor C (activator 1) 1, 145kDa
	RFC2	Replication factor C (activator 1) 2, 40kDa
	RFC4	Replication factor C (activator 1) 4, 37kDa
	RHBDF2	Rhomboid 5 homolog 2 (Drosophila)
	RNF146	Ring finger protein 146

	RNF168	Ring finger protein 168, E3 ubiquitin protein ligase
	RNF8	Ring finger protein 8, E3 ubiquitin protein ligase
	RPA1	Replication protein A1, 70kDa
	RUNX1	Runt-related transcription factor 1
	SDHAF2	Succinate dehydrogenase complex assembly factor 2
	SDHB	Succinate dehydrogenase complex, subunit B, iron sulfur (Ip)
	SETBP1	SET binding protein 1
	SETX	Senataxin
	SHPRH	SNF2 histone linker PHD RING helicase, E3 ubiquitin protein ligase
	SLX4	SLX4 structure-specific endonuclease subunit homolog (S. cerevisiae)
	SMAD4	SMAD family member 4
	SMARCA4	SWI/SNF related, matrix associated, actin dependent regulator of chromatin, subfamily a, member 4
	SMARCB1	SWI/SNF related, matrix associated, actin dependent regulator of chromatin, subfamily b, member 1
	SMARCE1	SWI/SNF related, matrix associated, actin dependent regulator of chromatin, subfamily b, member 1
	STK11	Serine/threonine kinase 11
	SUFU	Suppressor of fused homolog (Drosophila)
	TCL1A	T-cell leukemia/lymphoma 1A
	TELO2	TEL2, telomere maintenance 2, homolog (S. cerevisiae)
	TERF2	Telomeric repeat binding factor 2
	TERT	Telomerase reverse transcriptase
	TLR2	Toll-like receptor 2
	TLR4	Toll-like receptor 4
	TMEM127	Transmembrane protein 127
	TOPBP1	Topoisomerase (DNA) II binding protein 1
	TP53	Tumor protein p53
	TP53BP1	Tumor protein p53 binding protein 1
	TSC1	Tuberous sclerosis 1
	TSC2	Tuberous sclerosis 2
	TSHR	Thyroid stimulating hormone receptor
	UBE2A	Ubiquitin-conjugating enzyme E2A
	UBE2B	Ubiquitin-conjugating enzyme E2B

	UBE2I	Ubiquitin-conjugating enzyme E2I
	UBE2V2	Ubiquitin-conjugating enzyme E2 variant 2
	UBE4B	Ubiquitination factor E4B
	UIMC1	Ubiquitin interaction motif containing 1
	VHL	Von Hippel-Lindau tumor suppressor, E3 ubiquitin protein ligase
	WRN	Werner syndrome, RecQ helicase-like
	WT1	Wilms tumor 1
	XPA	Xeroderma pigmentosum, complementation group A
	XPC	Xeroderma pigmentosum, complementation group C
	XRCC1	X-ray repair complementing defective repair in Chinese hamster cells 1
	XRCC2	X-ray repair complementing defective repair in Chinese hamster cells 2
	XRCC3	X-ray repair complementing defective repair in Chinese hamster cells 3
	XRCC4	X-ray repair complementing defective repair in Chinese hamster cells 4
	XRCC5	X-ray repair complementing defective repair in Chinese hamster cells 5
	XRCC6	X-ray repair complementing defective repair in Chinese hamster cells 6
	ZNF350	Zinc finger protein 350
	ZNF365	Zinc finger protein 365
Breast and ovary cancers	BRCA1	BRCA1, DNA repair associated
	BRCA2	BRCA2, DNA repair associated
	BRIP1	BRCA1 interacting protein C-terminal helicase 1
	ATM	ATM serine/threonine kinase
	BARD1	BRCA1 associated RING domain 1
	CDH1	Caderina 1
	CHEK2	checkpoint kinase 2
	NBN	Nibrin
	NF1	Neurofibromin 1
	MRE11A	MRE11 homolog, double strand break repair nuclease
	PALB2	Partner and localizer of BRCA2
	PTEN	Phosphatase and tensinhomolog
	RAD50	RAD50 double strand break repair protein
	STK11	serine/threonine kinase 11
	AKT1	AKT serine/threonine kinase 1
	TP53	Tumor protein p53

	RAD51C	RAD51 paralog C
	RAD51D	RAD51 paralog D
	RAD51	RAD51 recombinase
	FAM175A	abraxas 1, BRCA1 A complex subunit - ABRAXAS1
	AKT1	AKT serine/threonine kinase 1
	FANCC	Fanconi anemia complementation group C
	MUTYH	mutY DNA glycosylase
	PIK3CA	phosphatidylinositol-4,5-bisphosphate3-kinase catalyticsubunitalpha
	RINT1	RAD50-Interacting protein 1
	SDHB	succinate dehydrogenase complex iron sulfur subunit B
	SDHD	succina tedehydrogenase complex subunit D
	XRCC2	X-rayrepair cross complementing 2
	BMPR1A	Bone morphogenetic protein receptor type 1A
	CDKN2A	Cyclin dependent kinase inhibitor 2A
	EPCAM	epithelial cella dhesion molecule
	MLH1	mutL homolog 1
	MSH2	mutS homolog 2
	MSH6	mutS homolog 6
	EPCAM	epithelial cell adhesion molecule
	PMS2	PMS1 homolog 2, mismatch repair system component
	SMAD4	SMAD family member 4
	VHL	von Hippel-Lindau tumor suppressor
	POLD1	DNA polymerase delta 1, catalytic subunit
	POLE	DNA polymerase epsilon, catalytic subunit
	AXIN2	axin 2
	CDK4	cyclin dependent kinase 4
Colon cancers	BMPR1A	bone morphogenetic protein receptor type 1A
	EPCAM	epithelial cell adhesion molecule
	PLA2G2A	Fosfolipasi A2
	NRAS	Neuroblastoma Ras viral oncogene homolog
	ODC1	Ornitina decarbossilasi 1
	CTNNB1	Catenina beta 1
	PIK3CA	Phosfatidylinositol 3 kinasi, catalytic alfa
	FGFR3	Fibroblast Growth Factor Receptor 3
	TLR2	Toll-likereceptor 2
	APC	adenomatous polypotus coli
	MCC	Mutated in colorectal cancer

	PTPN12	Protein-tyrosin fosfatasi, non receptor-type 12
	DLC1	deleted in liver cancer 1
	PDGFRL	Platelet-derived growth factor receptor-like
	RAD54B	ATRX, chromatin remodeler
	PTPRJ	Protein-tyrosine phosphatase, receptor-type, J
	CCND1	Cyclin D1
	MLH3	mutL homolog 3
	AKT1	AKT serine/threonine kinase 1
	BUB1B	BUB1 mitotic checkpoint serine/threonine kinase B
	TP53	Tumor protein 53
	FLCN	Folliculin
	AXIN2	Axisinhibitor 2
	DCC	deleted in colorectal carcinoma
	BAX	BCL2-Associated X protein
	SRC	SRC proto-oncogene, non-receptor tyrosine kinase
	AURKA	Aurora kinase A
	EP300	E1A binding protein p300
	PTEN	Phosphatase and tensin homolog
	CDH1	Caderina 1
	BRCA1	BRCA1, DNA repair associated
	BRCA2	BRCA2, DNA repair associated
	GREM1	Gremlin 1
	SMAD4	SMAD family member 4
	STK11	Serine/threonine kinase 11
	EPCAM	Epithelial cell adhesion molecule
	MLH1	mutL homolog 1, colon cancer, nonpolyposis type 2 (E. coli)
	MSH2	mutS homolog 2, colon cancer, nonpolyposis type 1 (E. coli)
	MSH6	mutS homolog 6 (E. coli)
	MUTYH	mutY homolog (E. coli)
	PMS2	Mismatch repair endonuclease PMS2
	POLD1	Polymerase (DNA directed), delta 1, catalytic subunit
	POLE	Polymerase (DNA directed), epsilon, catalytic subunit
	AKT1	RAC-alpha serine/threonine-protein kinase
	KLLN	Killin
	ATM	Ataxia telangiectasia mutated

	CHEK2	Checkpoint Kinase 2
	EXO1	Exonuclease 1
	GALNT12	Polypeptide N-acetylgalactosaminyltransferase 12
	PTPRJ	Protein Tyrosine Phosphatase, Receptor Type J
Multiple Endocrine neoplasms, Feocromocitomas-Paragangliomas	AIP	Aryl hydrocarbon receptor interacting protein
	BRAF	B-Raf proto-oncogene, serine/threonine kinase
	CDC73	cell division cycle 73
	CDKN1B	cyclindependent kinase inhibitor 1B
	GNAS	GNAS complex locus
	HRAS	HRas proto-oncogene, GTPase
	MEN1	HRas proto-oncogene, GTPase
	NF1	neurofibromin 1
	VHL	von Hippel-Lindau tumor suppressor
	MAX	MYC associated factor X
	GPR101	G protein-coupled receptor 101
	PRKAR1A	protein kinase cAMP-dependent type I regulatory subunit alpha
	RET	ret proto-oncogene
	SDHA	succinate dehydrogenase complex flavoprotein subunit A
	SDHAF2	succina tedehydrogenase complex assembly factor 2
	SDHB	succinate dehydrogenase complex iron-sulfur subunit B
	SDHC	succinate dehydrogenase complex subunit C
	SDHD	succinate dehydrogenase complex subunit D
	TMEM127	transmembrane protein 127
	EPAS1	endothelial PAS domain protein 1
	ATRX	ATRX, chromatin remodeler
	DAXX	death domain associated protein
	CDKN1A	cyclin dependent kinase inhibitor 1A
	CDKN2A	cyclin dependent kinase inhibitor 2A
	CDKN2B	cyclin dependent kinase inhibitor 2B
	CDKN2C	cyclin dependent kinase inhibitor 2C
	ESR2	estrogen receptor 2
	FH	fumarate hydratase
	FOXE1	forkhead box E1
	HBP2	hyaluronan binding protein 2
	KIF1B	kinesin family member 1B
	MAX	MYC associated factor X

	MDH2	malate dehydrogenase 2
	MINPP1	multiple inositol-polyphosphate phosphatase 1
	NTRK1	neurotrophic receptor tyrosine kinase 1
	IPMK	inositol polyphosphate multikinase
	EGLN1	prolyl hydroxylase domain-containing protein 2
	EGLN2	prolyl hydroxylase domain-containing protein 1
	MET	tyrosine-protein kinase Met
	FGFR1	fibroblast growth factor receptor 1
	H3F3A	histone H3.3
	IDH2	isocitrate dehydrogenase [NADP], mitochondrial
	KMT2D	histone-lysine N-methyltransferase 2D
	MERTK	proto-oncogene tyrosine-protein kinase MER
	TP53	Tumor protein p53
Melanoma	ACD	ACD, shelterin complex subunit and telomerase recruitment factor
	BAP1	BRCA1 associated protein 1
	CDK4	cyclin dependent kinase 4
	CDKN2A	cyclin dependent kinase inhibitor 2°
	CDKN2B	cyclin dependent kinase inhibitor 2B
	CDKN2D	cyclin dependent kinase inhibitor 2D
	MC1R	melanocortin 1 receptor
	MGMT	O-6-methylguanine-DNA methyltransferase
	MITF	melanogenesis associated transcription factor
	POT1	protection of telomeres 1
	TERF2IP	Telomeric repeat-binding factor 2-interacting protein 1
	TERT	telomerase reverse transcriptase
Lung cancer	DICER1	dicer 1, ribonuclease III
	RB1	RB transcriptional corepressor 1
	TP53	tumor protein p53
	TP73	tumor protein p73

The gene carrying the variant is indicated in bold.
